# Persistent Humoral Immune Responses in the CNS Limit Recovery of Reactivated Murine Cytomegalovirus

**DOI:** 10.1371/journal.pone.0033143

**Published:** 2012-03-06

**Authors:** Manohar B. Mutnal, Shuxian Hu, James R. Lokensgard

**Affiliations:** Neuroimmunology Laboratory, Department of Medicine, Center for Infectious Diseases and Microbiology Translational Research, University of Minnesota, Minnesota, United States of America; National Institute of Allergy and Infectious Diseases - Rocky Mountain Laboratories, United States of America

## Abstract

**Background:**

Experimental infection of the mouse brain with murine CMV (MCMV) elicits neuroimmune responses that terminate acute infection while simultaneously preventing extensive bystander damage. Previous studies have determined that CD8^+^ T lymphocytes are required to restrict acute, productive MCMV infection within the central nervous system (CNS). In this study, we investigated the contribution of humoral immune responses in control of MCMV brain infection.

**Methodology/Principal Findings:**

Utilizing our MCMV brain infection model, we investigated B-lymphocyte-lineage cells and assessed their role in controlling the recovery of reactivated virus from latently infected brain tissue. Brain infiltrating leukocytes were first phenotyped using markers indicative of B-lymphocytes and plasma cells. Results obtained during these studies showed a steady increase in the recruitment of B-lymphocyte-lineage cells into the brain throughout the time-course of viral infection. Further, MCMV-specific antibody secreting cells (ASC) were detected within the infiltrating leukocyte population using an ELISPOT assay. Immunohistochemical studies of brain sections revealed co-localization of CD138^+^ cells with either IgG or IgM. Additional immunohistochemical staining for MCMV early antigen 1 (E1, m112–113), a reported marker of viral latency in neurons, confirmed its expression in the brain during latent infection. Finally, using B-cell deficient (Jh^−/−^) mice we demonstrated that B-lymphocytes control recovery of reactivated virus from latently-infected brain tissue. A significantly higher rate of reactivated virus was recovered from the brains of Jh^−/−^ mice when compared to Wt animals.

**Conclusion:**

Taken together, these results demonstrate that MCMV infection triggers accumulation and persistence of B-lymphocyte-lineage cells within the brain, which produce antibodies and play a significant role in controlling reactivated virus.

## Introduction

Human cytomegalovirus (CMV) is the most significant infectious cause of congenital anomalies of the central nervous system (CNS), such as microcephaly and periventricular calcification. CMV is also the most frequent opportunistic cerebral infection in acquired immunodeficiency syndrome (AIDS) patients, in whom it can cause encephalitis and encephalopathy. In immunocompetent patients, immune control mechanisms effectively prevent overt disease and terminate viral replication. However, ultimate clearance of the viral genome is not achieved. Instead, like other herpesviruses, CMV remains life-long at specific sites. During nonproductive, latent infection viral gene expression is minimized to a small subset of genes [1]. The productive viral replication cycle can be initiated from this latent infection and result in transient phases of virus shedding and recrudescent disease. In immunocompromised patients, recurrence of human CMV infection frequently leads to overt manifestations of disease [2].

Our previous studies have shown that subsequent to intracerebroventricular (i.c.v.) infection with murine CMV (MCMV), viral brain infection is predominant in cells that line the periventricular region. These periventricular cells were subsequently identified as nestin positive, neural stem cells [3], [4]. The infection spreads into the parenchyma only in the absence of an effective CD8 response [5]. Evidence suggests that neural stem cells which escape the lytic phase of infection may differentiate into neurons that express a latency-associated antigen MCMV E1 (m112–113) [6]. These studies utilized neonatal mice to show preferential expression of E1 in latently-infected neurons. Establishment of latency after clearance of acute infection and the potential to reactivate to infection are key features of herpesvirus pathogenicity [7].

Reports using the MCMV model have established the importance of CD8^+^ T-cells for control of primary infection [8], [9]. Likewise, previous studies from our laboratory have shown that CD8^+^ T-cells play an important role in controlling acute brain infection and these antigen-specific cells persist even during the absence of active viral replication [10]. A number of previous studies using various RNA viruses have also revealed the long-term presence of viral genomes as well as immune cells in the brain, particularly T and B-lymphocytes [11], [12], [13]. These studies have shown that B-cell deficient mice were unable to control viral clearance from cortical and hippocampal neurons [14], [15]. However, little is known about the presence and role of B-lineage cells during MCMV brain infection.

In other models, it has been demonstrated that humoral immune responses driven by B-lymphocyte lineage cells can persist in nonlymphoid tissues following inflammatory insults. Neuroborreliosis, neurosyphilis, subacute sclerosing panencephalitis, and multiple sclerosis are all characterized by CNS accumulation of B-lymphocyte lineage cells such as antibody secreting cells (ASC) and elevated immunoglobulin in cerebral spinal fluid [16], [17]. During experimental CNS infections by RNA viruses such as Sindbis, Semliki Forest, rabies, and neurotropic coronaviruses, ASC appear to play a local protective role [18], [19], [20], [21]. Despite several reports describing the vital role that antibodies play during MCMV infection in peripheral organs, it remains to be determined whether a microenvironment which fosters humoral immune responses in the brain is created following MCMV infection and whether this response plays a significant role in controlling viral infection. In the present study, we investigated the kinetics of B-lymphocyte lineage cell recruitment into the brain, their ability to produce virus-specific antibodies, and their role in controlling viral infection.

## Results

### B-cell recruitment into the brain

**Figure 1:**
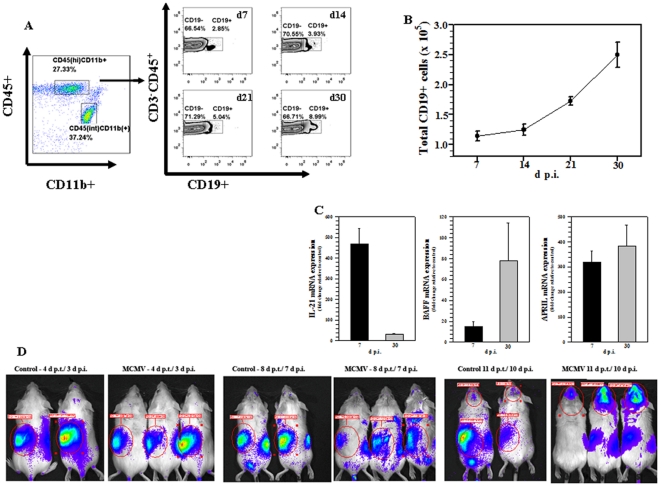
Entry and long-term persistence of B lymphocytes following MCMV brain infection. Single cell suspensions of brain tissue obtained from MCMV-infected mice (2–4 animals) per time point were banded on a 70% Percoll cushion. Brain leukocytes were collected and labeled with APC-conjugated Abs specific for CD45, Cy7-APC-labeled anti-CD11b, PE-CY7 anti-CD3, FITC anti-CD19 and analyzed using flow cytometry and FlowJo software. **A**. Representative FACS plots showing the percentages of CD45^+^CD19^+^ B lymphocytes within the infiltrating CD45^(hi)^CD3^−^ population within infected brains at 7, 14, 21 and 30 d p.i.. **B**. FITC-labeled anti-CD19 Abs were used to determine the total number of CD19^+^ B lymphocytes within the infiltrating CD45^(hi)^CD3^−^ population. Data shown are mean (±SEM) absolute number of infiltrating cells pooled from 3 independent experiments. **C**. mRNA levels for B-cell trophic factors were measured in total brain homogenates at the indicated time-points. **D**. Primed CD19^+^ B-cells from β-actin promoter-luciferase transgenic BALB/c mice were transferred via tail vein injection into MHC-matched, B-cell deficient (Jh^−/−^) recipients 1 d prior to viral infection. Representative dorsal bioluminescence images of 2 saline-injected controls and 3 infected recipient animals are shown at the indicated time-points.

Previous studies from our laboratory have shown that following MCMV brain infection, there is a clear distinction in the type of peripheral immune cell infiltration between the acute and chronic phases of disease. While innate components like neutrophils and macrophages constituted the majority of the cellular infiltrate during acute phase, persistence of antigen specific CD8^+^ T-cells was evident at 30 d post-infection (p.i.). In this study, multi-color flow cytometry was used to phenotype lymphocyte subpopulations infiltrating the brain at various time points p.i. Brain-infiltrating mononuclear cells were isolated on a percoll gradient as described in the methods. These isolated cells were subsequently immunostained using the markers CD45, CD11b, CD3 and CD19. B-cells expressing the CD19 marker were identified among the CD45^(hi)^CD11b^+^ population, which represent cells of myeloid origin. At 7 d p.i., 2.85% of the cells were reactive to CD19 and this population increased to 3.93%, 5.04%, and 8.99% at 14, 21 and 35 d p.i., respectively (Fig. 1A). The absolute number of CD19^+^ cells was also determined. An increase in the number of these cells was apparent starting at 7 d p.i. (1.1×10^5^±9.6×10^3^), 14 d p.i. (1.2×10^5^±8.5×10^3^), 21 d p.i. (1.7×10^5^±6.4×10^3^), until 30 d p.i. (2.4×10^5^±2.1×10^4^) (Fig. 1B).

Cytokines and B-cell survival factors play important role in recruitment and retention of B-lymphocyte lineage cells in the brain [13]. For this reason, we investigated the expression levels of IL-21, a key cytokine implicated in B cell proliferation and differentiation, which is predominantly secreted by CD4^+^ T cells [22]; B cell-activating factor of the tumor necrosis factor (TNF) family (BAFF); and a proliferating-inducing ligand (APRIL), both of which are considered to be B-cell lineage activating and survival factors [23], [24]. mRNA expression for IL-21 in infected brains was markedly higher than uninfected controls at 7 d p.i. (∼400 fold) and at 30 d p.i., its expression was still detectable (∼20 fold; Fig. 1C). Correspondingly, mRNA levels for BAFF and APRIL were also found to be elevated at 7, as well as 30 d p.i. Identifying these factors at the time of B-cell presence suggests that survival factors which play a crucial role in B lymphocyte retention are present following MCMV brain infection (Fig. 1C).

To visualize this B-cell trafficking into the brain, we went on to perform adoptive transfer experiments. Using a bioluminescence imaging approach, we transferred MCMV-primed CD19^+^ cells from β-actin promoter-luciferase expressing transgenic mice into B-cell deficient mice (Jh^−/−^). Jh^−/−^ recipient animals were given 5×10^6^ primed CD19^+^ cells 1 d prior to viral infection. The kinetics of CD19^+^ lymphocyte infiltration into the brain was examined longitudinally. Within 2 h of adoptive transfer, luciferase-positive cells were detected in the spleen. Movement of these CD19^+^ cells into the brain was observed at 10 d p.i. in Jh^−/−^ mice (Fig. 1D).

### Presence of plasma cells in the MCMV infected brain

**Figure 2:**
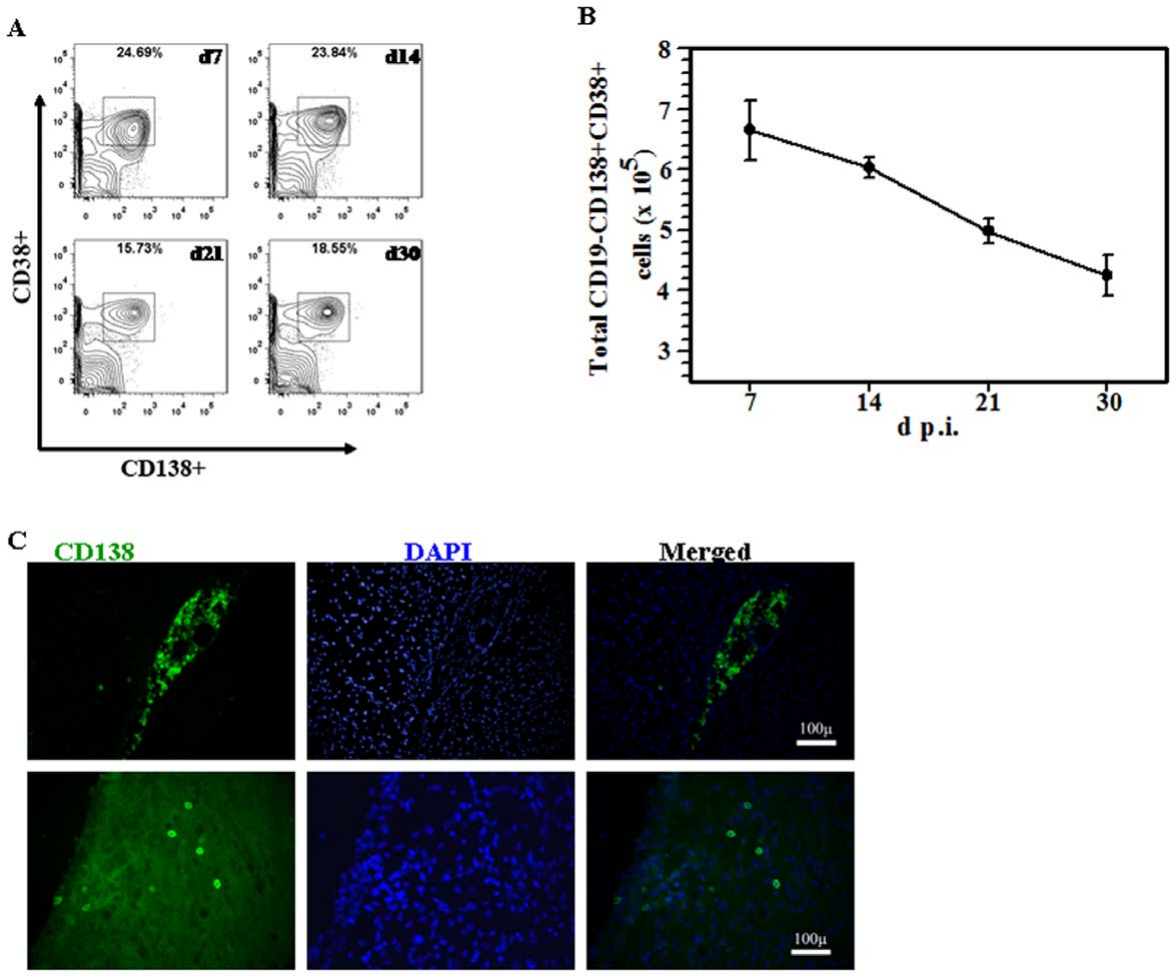
Plasma cell recruitment and retention in the brain following MCMV infection. **A**. Single cell suspensions of infiltrating brain leukocytes from MCMV-infected mice were obtained and analyzed at the indicated time points. Contour plots depicting percentages of CD38^+^ and CD138^+^ double positive cells from the gated CD45hi population are shown. These cells also stained negative for CD3 and CD19. **B**. Data showing the mean (±SEM) absolute number of cells within the infiltrating CD45^(hi)^CD3^−^CD19^−^ population pooled from 3 independent experiments. **C**. Immunofluorescent staining showing the distribution of CD138^+^ plasma cells in both the ventricles and brain parenchyma at 30 d p.i. (magnification 20×).

Following the detection of CD19^+^ cells in infected brains, we assessed the presence of CD19^−^CD138^+^CD38^+^ plasma cells among the brain-infiltrating mononuclear cells. Cells isolated on percoll gradients from infected brain tissue at various time points were stained for CD45, CD11b, CD3, CD19, CD38 and CD138. Those that expressed CD45^(hi)^CD3^−^CD19^−^CD138^+^CD38^+^ were phenotyped as plasma cells. Presence of these cells in the brain was observed at all time points studied, ranging from 15.73%–24.69% (Fig. 2A). The absolute number of CD19^−^CD138^+^CD38^+^ cells was found to peak at 7 d p.i. (6.6×10^5^±5.0×10^4^) and gradually decline at the later time points studied, 14, 21 and 30 d p.i. (5.9×10^5^±1.7×10^4^, 5.0×10^5^±2.1×10^4^ and 4.1×10^5^±3.3×10^4^, respectively) (Fig. 2B). The presence of plasma cells in the brain was also confirmed by immunohistochemical staining. Serial coronal sections were collected from infected brains at 30 d p.i. and were stained using Mabs against CD138. Persisting CD138^+^ cells were detected around the ventricles (Fig. 2C, upper panel), as well as within the cortex (Fig. 2C, lower panel).

### Long-term persistence of antibody secreting cells

**Figure 3:**
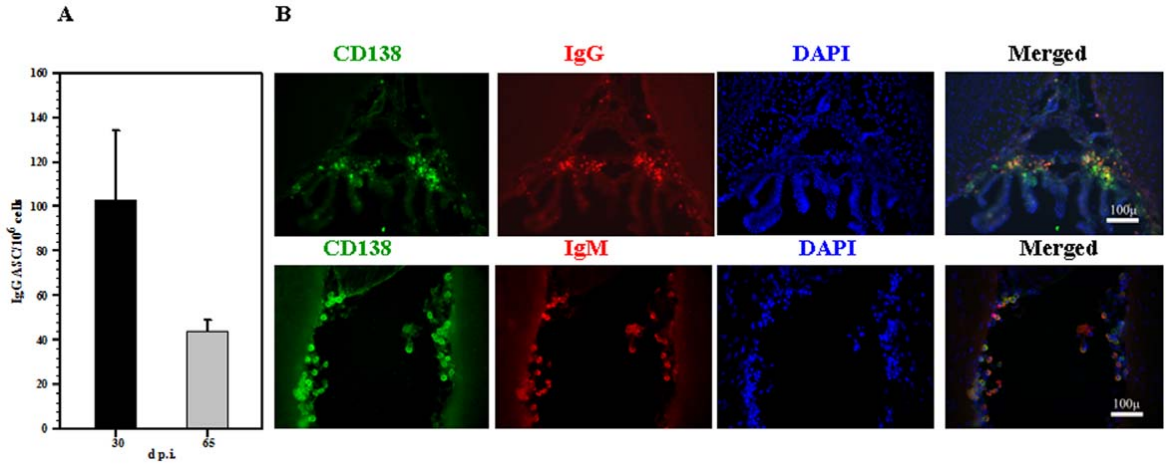
CD138^+^ ASCs persisting in the CNS produce antiviral antibodies. **A**. Frequencies of MCMV-specific ASCs persisting in the brain at 30 and 65 d p.i. as determined using ELISPOT. Data are presented as mean ± SD of triplicates and are from two independent experiments. **B**. Immunofluorescent staining of brain sections obtained from infected animals at 30 d p.i. for IgG and IgM co-localized with CD138 (magnification 20×).

Phenotypic analysis demonstrated that CD138^+^ plasma cells persisted in MCMV-infected brains. In order to investigate whether they were driving MCMV-specific humoral immune responses, ASCs isolated from infected brains were subjected to ELISPOT assay to detect MCMV-specific IgG production. At 30 d p.i., IgG-producing ASCs specific for MCMV were detected in infected brains (103±31 IgG ASC/10^6^ cells). The presence of ASCs was still evident, at 65 d p.i. (44±5 IgG ASC/10^6^ cells) (Fig. 3A). To determine the tissue distribution of CD138^+^IgG^+^ or CD138^+^IgM^+^ cells, we performed immunofluorescence staining. Four weeks p.i., sections were stained for CD138^+^, IgG, and IgM expressing cells. In these studies, CD138^+^ cells were found to be co-localized with either IgG or IgM and were predominantly detected in the subventricular zone, where MCMV actively replicates during acute phase of the disease (Fig. 3B), but double-positive cells were found in various brain regions including the cortex.

### Presence of latency-associated viral antigen

**Figure 4:**
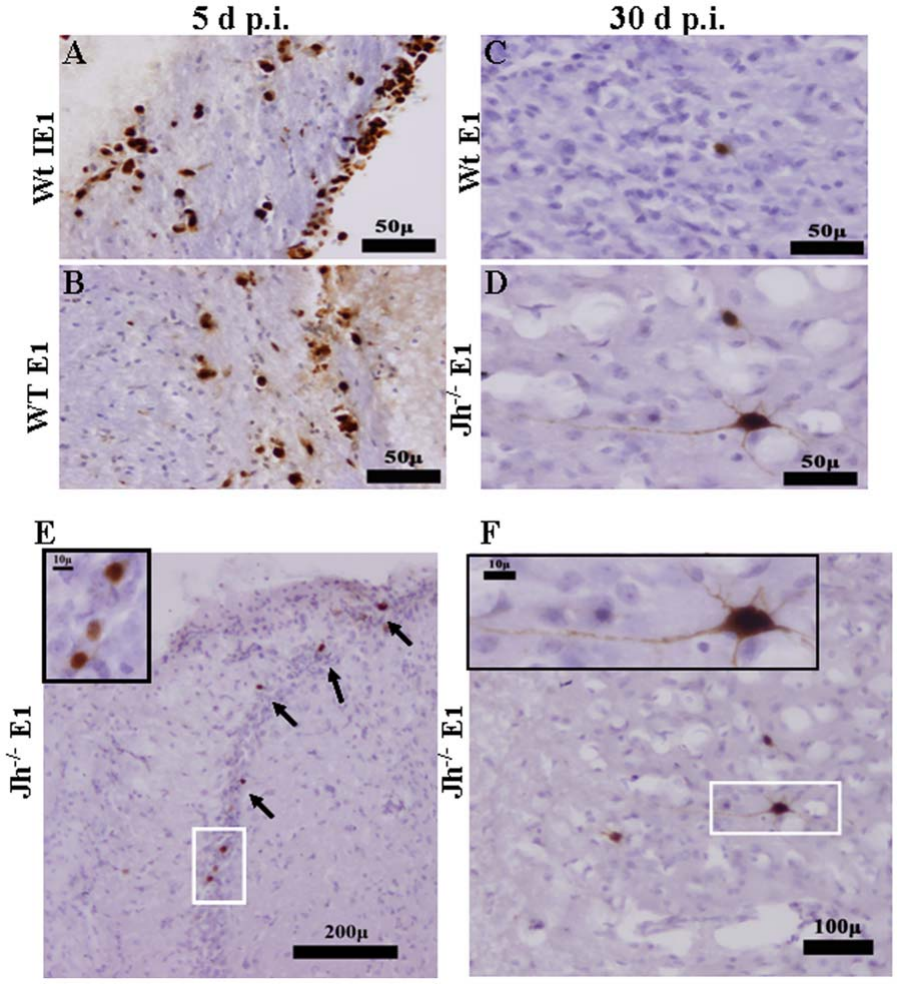
Latently-infected neurons expresses MCMV early 1 (E1, m112–113), but not immediate early 1 (IE1) antigen. Coronal murine brain sections were prepared and stained for viral proteins at indicated time points. **A**. Detection of MCMV IE1 and **B**. E1antigens in wild-type mice during acute infection (5 d p.i.), lower magnifications (20×) demonstrate that MCMV is localized to cells surrounding the ventricles. **C–F**. Tissue slices from infected brains were stained with an antibody to MCMV E1 during chronic infection (30 d p.i.). Cells positive for E1 antigen in the cortex and hippocampus of wild-type (**C**), as well as B-cell-deficient (Jh^−/−^) animals (**D, E and F**), are shown. Micrographs at higher magnification (40×) demonstrate the neuronal morphology of infected cells.

Previously, we have reported that active viral expression is not detected at 30 d p.i., either by immunohistochemical staining for MCMV-IE1 in brain tissue sections, or by mRNA real-time PCR for MCMV-IE1 genome in total brain homogenates. Earlier reports, using neonatal mice, suggested that the MCMV early gene E1 (M112–113) product has a tendency to be retained in neurons following MCMV-infection [25], especially during prolonged infection [26]. These studies suggested that neurons contain factors that drive promoter expression for this MCMV early gene in the absence of viral immediate-early (IE) gene products. In the present study, using adult mice, we confirmed that MCMV-E1 expression does occur in neurons within latently-infected brains. IE1 and E1 expression were both evident at 5 d p.i. (Fig. 4 A, B), whereas only E1 expression was observed at 30 d p.i., in both Wt and Jh^−/−^ mice (Fig. 4, C–F).

### B-lineage cells limit recovery of reactivated virus

**Figure 5:**
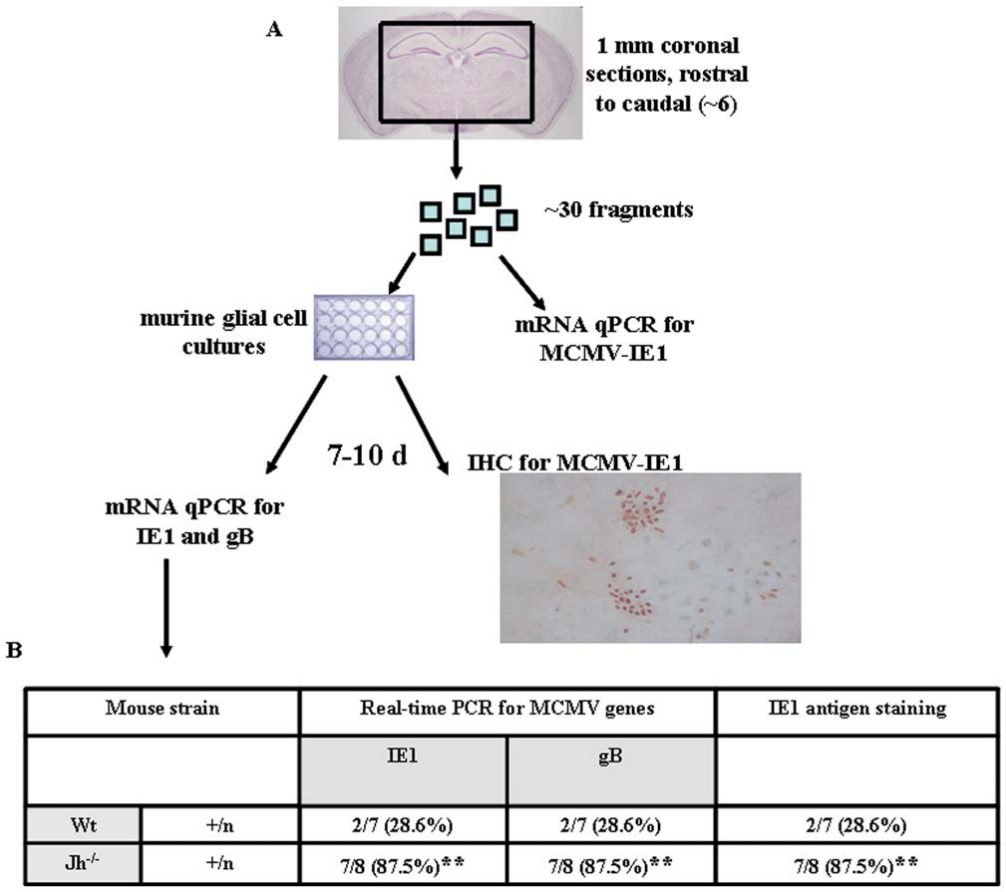
Reactivated infectious virus is recovered more efficiently from the brains of B-cell deficient animals. **A**. Periventricular brain tissue obtained from latently-infected animals (>30 d p.i.) was cut into 1 mm pieces and placed onto primary murine glial cell cultures. At 10 d post-explant, these cultures were stained for MCMV-IE1 expression, indicative of viral reactivation, and processed for real-time PCR (MCMV IE1 and gB genes). **B**. Explant reactivation results in Wt versus Jh^−/−^ mice as determined using real-time PCR for IE1 and gB, as well as IE1 antigen staining. Data are shown as the number positive animals over total number examined (+/n). **p<0.01 Jh^−/−^ versus Wt animals.

Following the demonstration of latent viral protein in infected brain sections, we then designed experiments to reactivate the virus. Previous studies using an Ig μ chain–deficient mouse, devoid of B-cells and antibodies, revealed that antibodies are not required for the resolution of primary MCMV infection, and latency is established with the same kinetics as in seropositive controls [27], [28]. Using this model, studies have shown that virus could be reactivated in various organs including, salivary glands, lungs and spleen [29]. These findings suggested that B-cells, along with other immune cells, play a vital role in preventing the recovery of reactivated virus from different organs. However, the role of these cells in the brain was not addressed. Using a similar mouse strain, Jh^−/−^, which fails to produce functional B-cells as they lack the gene for the heavy chain joining region, we investigated the effect of B-lineage cells on recovery of virus reactivated from latently infected brain tissue. Latently infected brain tissue samples were collected from Wt and Jh^−/−^ and were processed as outlined in the Fig. 5A. Slow-dividing, primary murine glial cells were used as indicator cells for explant cultures. In addition, direct homogenized tissue samples collected at 30 d p.i. were tested negative for MCMV-IE1 genes in Wt and Jh^−/−^ mice by real-time PCR assay, indicating an absence of productive viral infection. 7–10 d post–explant, murine mixed glial cultures were either stained for the presence of MCMV-IE1 protein by immunohistochemistry or prepared for real-time PCR. In these experiments, recovery of reactivated virus was found to be significantly higher in the Jh^−/−^ B-cell deficient mice, 7/8 (87%), when compared to Wt animals, 2/7 (28.6%), as detected by the presence of viral gene products in the explant cultures (Fig. 5B).

## Discussion

Although several studies have demonstrated humoral immune responses in the CNS following infectious or inflammatory assault, the persistence of B-lymphocyte lineage cells in the brain following MCMV infection has not been reported. Our results demonstrated B-cell recruitment into the CNS occurs early in the disease and persists during latent infection. In addition, ASCs isolated from brain were tested for MCMV-specific antibody production and the significance of these cells in controlling infectious virus was demonstrated using B-cell deficient animals.

The accumulation and retention of virus-specific ASC within the CNS are hallmarks of many neurotropic viral infections. Several studies have characterized the cytokines and chemokines essential for recruitment and survival of T- and B-cells in the CNS. In this study, we detected expression of B-cell trophic factors such as IL-21, APRIL and BAFF. IL-21 is a type I cytokine that signals through a receptor composed of IL-21R and the common cytokine receptor γ-chain [30]. Over the past decade, IL-21 has emerged as a key cytokine for the differentiation of activated B lymphocytes into plasma cells and, in consequence, for establishing and maintaining Ab responses. IL-21 is produced mainly by distinct T lymphocyte subsets. It has been established in studies using human cells that mature plasma cells do not express IL-21 receptors, hence do not require IL-21 [22]. In our study, decreased levels of IL-21 in the brain were observed at 30 d.p.i., when a higher number of ASCs was detected. Similar findings have been reported during other viral brain infections [13].

Previous studies from our laboratory utilizing HSV [31] and MCMV [10], [32] brain infection models have shown that T-cells play a vital role in controlling infections in this immunologically privileged site. Entry of peripheral immune cells into the brain is essential for clearance of infectious virus [33] and previous reports using RNA viruses have shown that IFN-γ and anti-viral IgG are required for this effect [14], [21]. We have previously demonstrated the accumulation and retention of virus-specific T-cells in the brain following MCMV infection and these long-term persisting cells were found to produce IFN-γ upon ex-vivo stimulation with viral peptides [10]. The recruitment of B-lineage cells into brain has been shown to be dependent upon cytokine and chemokine responses generated by infiltrating T-cells, possibly mediated through CXCR3 [34].

Due to the inherent nature of herpesviruses to establish latency it is imperative to identify host factors that restrict virus reactivation in this vital organ. The role of B-cells has been well established in restricting MCMV in other organs including lungs, spleen and salivary glands [29], but their role in restricting virus reactivation in the brain is unclear. B-lineage cells were detected in the brain as early as 7 d p.i. Similar observations have been made in other viral brain infections where a surprising number of CD138^+^ cells at 7 d p.i. has been reported [35]. When we assessed the infiltrating immune cells for production of virus-specific antibodies by ELISPOT assay, these virus-specific ASCs were detected for as long as 65 d p.i. Similar trafficking and persistence of ASC in the CNS has been reported in other RNA virus models such as West Nile virus and mouse Alpha-virus infections [12], [35].

Similar to other members of the herpesviridae, CMVs remain within their host and establish lifelong latency after primary infection. Depending on the host and viral system, the question of whether the virus is truly latent or maintains a low-level, persistent viral infection has not been completely resolved. Operationally, latency is defined as the inability to detect replicating virus despite the presence of virus DNA and perhaps some limited transcription. In murine models of MCMV infection, latency is characterized by the ability to reactivate virus from cells of infected tissues after cocultivation with permissive cells in culture, even though infectious virus cannot be detected directly in tissue homogenates [36], [37]. Immunosuppression [38] or immunologic modulation, such as allogeneic stimulation [39], has been shown to induce reactivation of latent virus. Results of the studies reported here support the idea that after MCMV brain infection, virus becomes latent in neurons and that this latent virus may subsequently reactivate. In addition to the brain, results from several studies suggest that MCMV also becomes latent in other organs, including eye [38], lungs [40] and salivary glands [41]. Earlier studies have also demonstrated that virus recovery from other latently infected tissues, such as salivary gland and lungs, is 100–1000 fold higher in B-cell deficient mice [29].

Previous reports have suggested that the early gene E1 product is retained in neurons of MCMV-infected mouse brains [25], especially during prolonged infection [26], and could, therefore, be used as a marker of viral latency. These studies utilized neonatal infection models to demonstrate differential expression of various gene products in diverse cellular sources. In this study, utilizing the same monoclonal antibody, we confirmed that E1 expression was evident in the hippocampal region and, based upon the morphology of infected cells, it appears that neurons were the cell type harboring latent viral infection. Interestingly, we failed to detect mRNA for the E1 gene directly from brain homogenate at 30 d p.i., possibly due to low copy numbers and detection limits in the homogenates.

Results presented here demonstrate that humoral immune responses in the CNS are important in controlling reactivated virus. Jh^−/−^ mice had a higher frequency of virus recovery from the brain, suggesting that B-cells directly limit viral recovery. Taken together these results suggest that MCMV infection results in the recruitment of B-lineage cells into the brain and these cells play important role in controlling reactivated virus.

## Methods

### Ethical statement

This study was carried out in strict accordance with recommendations in the Guide for the Care and Use of Laboratory Animals of the National Institutes of Health. The protocol was approved by the Institutional Animal Care and Use Committee (Protocol Number: 0807A40181) of the University of Minnesota. All surgery was performed under Ketamine anesthesia, and all efforts were made to minimize suffering.

### Virus and animals

RM461, a MCMV expressing *Escherichia coli* β-galactosidase under the control of the human ie1/ie2 promoter/enhancer [42] was kindly provided by Edward S. Mocarski. The virus was maintained by passage in weanling female BALB/c mice. Salivary gland-passed virus was then grown in NIH 3T3 cells for 2 passages, which minimized any carry over of salivary gland tissue. Infected 3T3 cultures were harvested at 80% to 100% cytopathic effect and subjected to three freeze–thaw cycles. Cellular debris was removed by centrifugation (1000×*g*) at 4°C, and the virus was pelleted through a 35% sucrose cushion (in Tris-buffered saline [50 mM Tris–HCl, 150 mM NaCl, pH 7.4]) at 23,000×*g* for 2 h at 4°C. The pellet was resuspended in Tris buffered saline containing 10%FBS. Viral stock titers were determined on 3T3 cells as 50% tissue culture infective doses (TCID_50_) per milliliter. Six to eight weeks old BALB/c mice were obtained from Charles River Laboratories (Wilmington, MA), while B-cell deficient mice (Jh^−/−^) were a kind gift from Dr. Steven McSorley (University of California, Davis).

### Intracerebroventricular infection

Infection of mice with MCMV was performed as previously described [5]. Briefly, female mice (6–8 week old) were anesthetized using a combination of Ketamine and Xylazine (100 mg and 10 mg/kg body weight, respectively) and immobilized on a small animal stereotactic instrument equipped with a Cunningham mouse adapter (Stoelting Co., Wood Dale, IL). The skin and underlying connective tissue were reflected to expose reference sutures (sagittal and coronal) on the skull. The sagittal plane was adjusted such that the bregma and lambda were positioned at the same coordinates on the vertical plane. Virulent, salivary gland-passaged MCMV RM461 (1.5×10^5^ TCID_50_ units in 10 µl), was injected into the right lateral ventricle at 0.9 mm lateral, 0.5 mm caudal to the bregma and 3.0 mm ventral to the skull surface using a Hamilton syringe (10 µl) fitted to a 27 G needle. The injection was delivered over a period of 3–5 min. The opening in the skull was sealed with bone wax and the skin was closed using 9 mm wound clips (Stoelting Co., Wood Dale, IL).

### Isolation of brain leukocytes and FACS

Leukocytes were isolated from MCMV-infected murine brains using a previously described procedure with minor modifications [21], [43], [44], [45]. In brief, brain tissues harvested from four to six animals were minced finely in RPMI 1640 (2 g/L D-glucose and 10 mM HEPES) and digested in 0.0625% trypsin (in Ca/Mg-free HBSS) at room temperature for 20 min. Single cell preparations from infected brains were resuspended in 30% Percoll and banded on a 70% Percoll cushion at 900× g at 15°C. Brain leukocytes obtained from the 30–70% Percoll interface were treated with Fc block (anti-CD32/CD16 in the form of 2.4G2 hybridoma culture supernatant with 2% normal rat and 2% normal mouse serum) to inhibit nonspecific Ab binding and were stained with anti-mouse immune cell surface markers for 45 min at 4°C (anti-CD45-allophycocyanin (eBioscience), anti-CD11b-FITC or anti-CD11b-APC-CY7, anti-CD19-FITC, anti-CD138-APC, anti-CD38-PE, and anti-CD3-PE-Cy7 (BD Biosciences)) and analyzed by flow cytometry. Control isotype Abs were used for all isotype and fluorochrome combinations to assess nonspecific Ab binding. Live leukocytes were gated using forward scatter and side scatter parameters on a BD FACSCanto flow cytometer (BD Biosciences). Data was analyzed using FlowJo software (TreeStar).

### Adoptive transfer

FVB/N luciferase transgenic mice (luciferase expression driven by the β-actin promoter; Xenogen) were backcrossed for 10 generations into BALB/c mice (Charles River Laboratories) and were bred in house. Luciferase activity of resulting BALB/c Luc^+^ mouse colony was confirmed for 5 generations prior to use. These BALB/c Luc^+^ mice were used as donors in adoptive transfer experiments. Spleens from MCMV primed (1×10^5^ TCID_50_/mouse, i.p. injection) donor animals were collected aseptically at 7 d post-priming. Single cell suspensions of immunocytes were depleted of RBC by treatment with 0.87% ammonium chloride, washed twice, and cell viability was confirmed using trypan blue. CD19^+^ lymphocytes were enriched by negative selection using a CD19^+^ cell purification kit, as per the manufacturer's instructions (R&D systems, Minneapolis, MN USA). Immune cells were transferred (5×10^6^ cells/mouse) via tail vein 1 d prior to infection with MCMV into syngenic (Jh^−/−^) recipients.

### Bioluminescence imaging

Imaging of firefly luciferase expression in live animals was performed using an IVIS50 (Xenogen) equipped with a charge-coupled camera device, as previously described with minor modifications [46]. In brief, 150 µg of D-luciferin (Gold Biotechnology) was administered to anesthetized mice by i.p. injection. Animals were imaged 5 min after D-luciferin administration and data were acquired using a 5-min exposure window. Bioluminescence imaging studies were conducted with age-matched 8- to 10-wk-old female Jh^−/−^ mice as recipients and MHC-matched female BALB/c luciferase transgenic mice as leukocyte donors. Immune cells were derived from the spleens of MCMV primed animals. The enriched CD19^+^ lymphocyte population was used for the adoptive transfer experiments.

### Immunohistochemistry to detect MCMV-E1 or IE1, CD138, IgG and IgM

Brains were harvested from infected mice that were perfused with serial washes of 2% sodium nitrate and phosphate-buffered saline (PBS) to remove contaminating blood cells and prefixed with 4% paraformaldehyde. Murine brains were subsequently submerged in 4% paraformaldehyde for 24 h and transferred to 30% sucrose solution for 2 d. After blocking (1× PBS, 10% normal goat serum and 0.3% Triton X-100) for 1 h at room temperature, brain sections (25 µm) were incubated overnight at 4°C with the following primary antibodies: rat anti-mouse CD138 (Syndecan-1, a plasma cell marker), (1∶100; BD Biosciences, Franklin Lakes, NJ), biotinylated goat anti-mouse IgG and Ig53M (1∶600; Jackson ImmunoResearch Laboratories, West Grove, PA), MCMV IE1-specific monoclonal antibody (MAb Croma 101, kindly provided by Dr. Stipan Jonjic, University of Rijeka, Croatia), monoclonal anti-E1 antibody (MAb kindly provided by Dr. I Kosugi, Hamamatsu University, Japan). Brain sections were washed three times with PBS and then incubated with fluorescein (FITC)–conjugated anti rat antibody (1∶200; Vector Laboratories, Burlingame,CA), or Cy3- conjugated streptavidin (1∶400; Jackson ImmunoResearch Laboratories) for 1 h at RT. Virus-specific antigen were detected using an immunoperoxidase method. After a 5 min pretreatment with 0.3% H_2_O_2_, a subset of sections from each animal was incubated with primary antibodies overnight at 4°C. The sections were then rinsed and incubated for 1 h in biotinylated goat anti-mouse (Vector Laboratories). The goat anti-mouse biotinylated antibody was reacted with an avidin-HRP conjugate. The HRP chromagen was developed using diaminobenzidine (DAB) substrate (Vector Laboratories). Sections were counterstained with Mayer's hematoxylin.

### Real-time PCR

Total RNA was extracted from brain tissue homogenates using the Trizol reagent (Invitrogen, Carlsbad, CA). One µg RNA was DNase (Ambion, Applied Biosystems, Austin, TX) treated and reverse transcribed to cDNA with SuperScript™ III (Invitrogen), dNTP (GE Healthcare, Piscataway, NJ) and oligo (dT)_12–18_ (Promega, Madison, WI). Real-time PCR was performed in an Mx3000p (Stratagene, La Jolla, CA) with SYBR Advantage qPCR Premix (Clontech, Mountain View, CA), primers and cDNA according to the manufacturer's protocol. Reaction conditions for qPCR were as follows: initial denaturation at 95°C for 15 sec, amplification for 40 cycles at 95°C for 10 sec, 60°C for 10 sec and 72°C for 10 sec followed by dissociation curve analysis (1 cycle at 95°C for 60 sec, 55°C for 30 sec and 95°C for 30 sec) to verify product specificity. After normalizing to HPRT-1 expression (ΔCt = target gene Ct−HPRT Ct) and then to the control group (ΔΔCt = treatment ΔCt−C ΔCt), relative quantification using 2∧^−ΔΔCt^ was calculated as fold change of target mRNA expression vs. control. Primer sequences for MCMV IE1 [10], mAPRIL, BAFF, and IL-21 [13] were obtained from published literature.

### ELISPOT assay for ASC

ELIPOT for ASC isolated from the brain was performed using previously published methods [35]. Briefly, plates (96-well nitrocellulose; Millipore, Billerica, MA) were coated with serum-free MCMV (1×10^6^ TCID_50_/well) or conditioned medium (irrelevant antigen) and then blocked with RPMI plus 10% FBS. Mononuclear cells were isolated from the brains of MCMV-infected mice as described above, added to wells (2×10^5^ cells/well) in triplicate, and incubated overnight. ASC spots were developed by sequential addition of biotinylated anti-IgG (Vector Laboratories, Burlingame, CA), horseradish peroxidase-conjugated strepavidin (Vector Laboratories), and 3-amino-9-ethyl carbazole substrate (BD Biosciences) and counted using a dissecting microscope. The numbers of specific ASC were determined by subtraction of the average number of spots in the irrelevant antigen wells (range: 0–5) from the average number of spots in the MCMV-specific antigen wells.

### Ex vivo reactivation assay

Brains were isolated from latently infected BALB/c and Jh^−/−^ mice and 1 mm coronal sections were cut using a precision brain matrix (Braintree Scientific, Braintree, MA, USA). The coronal sections were then cut around the ventricles; ∼25 pieces were collected from each brain that represented tissue adjoining lateral and central ventricles. 3–4 pieces were then inoculated onto previously prepared primary mouse brain cultures in a 12-well plate. The cultures were maintained in Dulbecco's modified Eagle's medium supplemented with 10% fetal calf serum and antibiotics. The cultures were analyzed microscopically for the occurrence of cytopathic effect. Half of the wells containing primary mouse brain culture cells infected with the recovered viruses were analyzed by immunostaining using monoclonal antibodies Croma101 (anti-MCMV IE1) and a secondary horseradish peroxidase-conjugated anti-mouse antibody (Dako, Hamburg, Germany). The remaining wells were used for RNA isolation and real-time PCR detection of MCMV-IE1 and gB gene expression.
